# Alcohol policy scores: data and analysis

**DOI:** 10.2471/BLT.14.150144

**Published:** 2015-08-31

**Authors:** John Duffy

**Affiliations:** a 15/4 Coltbridge Millside, Edinburgh, EH12 6AP, Scotland.

Carragher et al.^1^ describe the development and application of an alcohol policy score (TEASE-16) to nine study areas in the western Pacific. In their analysis they attempt to relate the policy score to average alcohol consumption for these areas. My analysis shows that their correction for income is not required, and that their use of division to correct for consumption needs justification, because it produces a consumption variable of litres per dollar.

In an attempt to analyse the cross-sectional data correctly, I obtained information on alcohol consumption for eight of the study regions from published WHO data for 2010,^2^ which does not separate data for Hong Kong Special Administrative Region from the rest of China. I obtained gross domestic product (GDP) and population data for 2010 from published World Bank information.

I did a multiple regression analysis using methods described by Kronmal^3^ to avoid the well-known problem of spurious correlation arising from the use of rates.^4^ The results show that alcohol consumption does not appear to be related to income (measured by GDP or GDP per-capita) or to TEASE-16 for the eight regions considered. However TEASE-16 is related to GDP, but not in a linear fashion.

In my regression model, I considered total alcohol consumption in each area as the response, with GDP, population and TEASE-16 score the predictors. Only population size was significantly associated with total alcohol consumption (Table 1), and examination of outliers show that this association arises because of the influence of China with its much larger population. Fitting of the submodels involving only population and TEASE-16 and only GDP and population did not alter this conclusion. Similarly, including total population aged over 15 as a predictor did not alter the conclusion.

**Table 1:** Multiple regression results for alcohol consumption with predictors GDP, TEASE-16 and population

Variable		Coefficients, beta (SE)	Standardized coefficients, beta	*P*
	Constant	298 255 637 (176 624 877)	–	0.167
	GDP	0.001 (0.00053)	0.193	0.135
	TEASE-16	3 527 087 (3 307 219)	0.019	0.346
	Population	4.698 (0.600)	0.811	0.001

GDP: gross domestic product; SE: standard error.

**Fig. 1:**
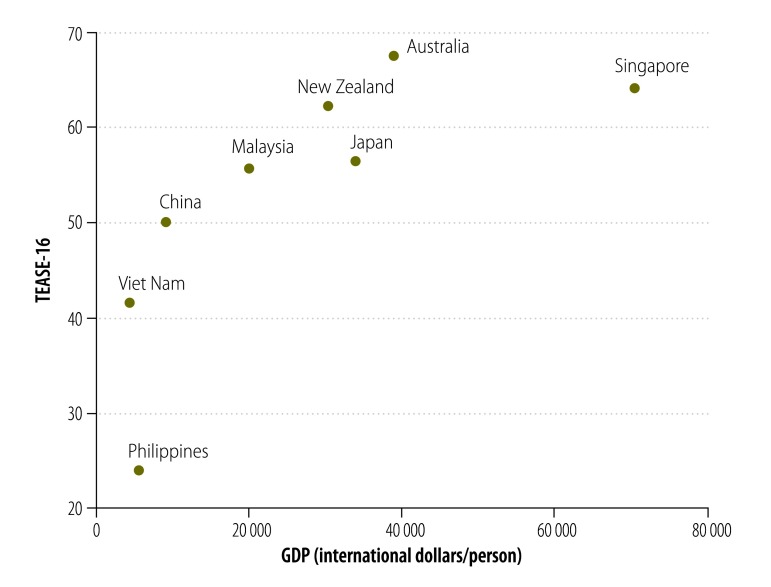
Relationship between TEASE-16 and GDP per capita in the western Pacific

The relationship between TEASE-16 and GDP per capita is shown in Fig. 1. It can be seen that the relationship is not linear and I confirmed this by fitting a regression model with a quadratic term in GDP. Fitting a regression relationship as in Table 1, but including the square of GDP as a predictor once again, did not alter the conclusions. The inappropriate adjustment done by Carragher et al. for income is the source of the spurious relationship between TEASE-16 and consumption, since TEASE-16 is positively related to income and the consumption scores are divided by income, resulting in a negative relationship that is an artefact of the analysis.

GDP: gross domestic product.

Neither TEASE-16 score nor income, (measured by GDP), were significantly correlated with alcohol consumption across the eight areas in the year 2010. It seems unlikely that analysis of the original data used by Carragher et al.^1^ would alter this conclusion (if so, it would raise concerns related to data quality), but I would be happy to undertake such analysis if the data were made available.
